# Multiple nutritional phenotypes of fission yeast mutants defective in genes encoding essential mitochondrial proteins

**DOI:** 10.1098/rsob.200369

**Published:** 2021-04-07

**Authors:** Lisa Uehara, Shigeaki Saitoh, Ayaka Mori, Kenichi Sajiki, Yusuke Toyoda, Fumie Masuda, Saeko Soejima, Yuria Tahara, Mitsuhiro Yanagida

**Affiliations:** ^1^ Okinawa Institute of Science and Technology Graduate University, Tancha 1919-1, Onna, Okinawa 904-0495, Japan; ^2^ Institute of Life Science, Kurume University, Asahi-machi 67, Kurume, Fukuoka 830-0011, Japan

**Keywords:** mitochondria, *ts* mutants, nutritional stress, ribosome, RNA processing, fatty acid synthesis

## Abstract

Mitochondria are essential for regulation of cellular respiration, energy production, small molecule metabolism, anti-oxidation and cell ageing, among other things. While the mitochondrial genome contains a small number of protein-coding genes, the great majority of mitochondrial proteins are encoded by chromosomal genes. In the fission yeast *Schizosaccharomyces pombe*, 770 proteins encoded by chromosomal genes are located in mitochondria. Of these, 195 proteins, many of which are implicated in translation and transport, are absolutely essential for viability. We isolated and characterized eight temperature-sensitive (*ts*) strains with mutations in essential mitochondrial proteins. Interestingly, they are also sensitive to limited nutrition (glucose and/or nitrogen), producing low-glucose-sensitive and ‘super-housekeeping' phenotypes. They fail to produce colonies under low-glucose conditions at the permissive temperature or lose cell viability under nitrogen starvation at the restrictive temperature. The majority of these *ts* mitochondrial mutations may cause defects of gene expression in the mitochondrial genome. *mrp4* and *mrp17* are defective in mitochondrial ribosomal proteins. *ppr3* is defective in rRNA expression, and *trz2* and *vrs2* are defective in tRNA maturation. This study promises potentially large dividends because mitochondrial quiescent functions are vital for human brain and muscle, and also for longevity.

## Graphical abstract

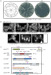

## 1. Introduction

In mitochondria, the cell organelle for respiration, pyruvate is enzymatically catabolized in the citric acid (TCA) cycle to H_2_O and CO_2_ by a complex series of electron-transfer, respiratory reactions, so as to produce ATP and NADH. The mitochondrial genome encodes only a small number of proteins and RNAs. However, mitochondrial functions also require orchestrated expression of many chromosomally encoded genes. Chromosomally encoded mitochondrial proteins perform diverse functions, such as protein transport and synthesis, which supports respiratory functions of mitochondria. In addition to cellular respiration, mitochondria participate in iron-sulfur cluster formation, metabolism of nutritional molecules (e.g. folate, fatty acids, amino acids and nucleotide), and apoptosis [1–4]. In humans, many diseases that impact brain and muscle functions are caused by malfunctioning mitochondria under increased oxidative stress, so full understanding of mitochondrial functions is important for human longevity [5,6].

The fission yeast *Schizosaccharomyces pombe* belongs to a group of ‘petite negative' yeasts, in which mitochondrial DNA is essential for viability, in contrast to the ‘petite positive' budding yeast *Saccharomyces cerevisiae*, which can survive without mitochondrial DNA [7–10]. Even in the presence of a respiratory poison (antimycin A), *S. pombe* cells can grow and divide at a reduced rate, if glucose concentrations are higher than 0.2% [11,12]. Respiratory activity thus appears to be optional for cell proliferation under conditions in which an adequate concentration of glucose is present, while it is important for synthesis of amino acids derived from the Krebs cycle metabolite α-ketoglutarate [11].

From a genome-wide analysis of essential genes, it was reported that the most striking difference between budding yeast, *S. cerevisiae*, and fission yeast, *S. pombe*, is mitochondrial function [13]. Many (96) *S. pombe* genes for mitochondrial protein translation machinery are essential (electronic supplementary material, table s1), compared with only 6 genes in budding yeast. The small mitochondrial genome [13] is similar to that of higher eukaryotic organisms. *Schizosaccharomyces pombe* mitochondria may thus be a convenient model, but studies of *S. pombe* chromosomal genes for mitochondrial proteins have been relatively scarce. Regarding the number of publications focusing on mitochondria, budding yeast studies are approximately 100-fold more numerous than those of *S*. *pombe*. Hence, a basic understanding of fission yeast mitochondria, comparable to that of budding yeast is highly desirable. In this study, we describe several *S. pombe* temperature-sensitive (*ts*) strains defective in mitochondrial functions, since such mutants are scarce for *S*. *cerevisiae*.

## 2. Results

### 2.1. Temperature-sensitive mutations affecting genes with annotated mitochondrial functions

A collection of approximately 1000 *S. pombe ts* strains previously constructed [14–16] was screened to identify genes responsible for temperature sensitivity. Responsible genes were identified using a classical forward-genetics approach. Minimal chromosomal fragments that suppress the *ts* phenotype were obtained by transformation, followed by nucleotide sequencing. Candidate genes were examined by tetrad dissection to determine whether they are genetically linked to the *ts* phenotype. This approach was time-consuming and inefficient because many chromosomal fragments obtained proved to contain high-copy suppressors rather than the responsible gene, and mitochondrial mutants were not enriched in the collection. After years of attempts, we succeeded in identifying 8 *ts* mutations in genes, products of which were implicated in mitochondrial functions and/or were localized to mitochondria.

**Figure 1.**
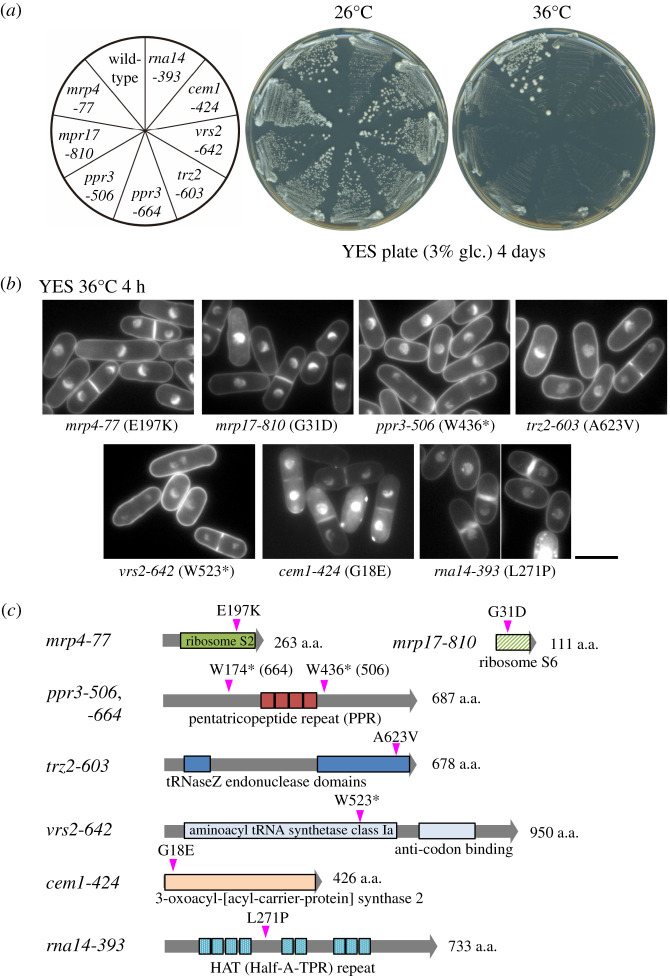
Isolation of eight *ts* mitochondrial strains. (*a*) Wild-type and eight *ts* strains in which a gene encoding a mitochondrial protein is mutated, are streaked on YES solid media and incubated at 26°C (permissive temperature) or 36°C (restrictive temperature) for 4 days. While all strains formed colonies at 26°C, *ts* strains failed to form colonies at 36°C. (*b*) Cellular and nuclear morphology was examined in *ts* mitochondrial strains using fluorescence microscopy. Cells of indicated mutant strains were cultivated in YES liquid medium at 36°C, and fixed with 2.5% glutaraldehyde. DNA of fixed cells was stained with DAPI, prior to microscopy. Scale bar, 10 µm. (*c*) Gene products responsible for phenotypes of the mutants are schematically depicted. Mutation sites are indicated by arrowheads (magenta), along with characteristic domains of each product. An asterisk represents a stop codon.

Products of the identified *ts* mutant genes are listed in table 1. Gene cloning and sequencing showed seven distinct *ts* mutant genes (*mrp4, mrp17, ppr3, trz2, vrs2, cem1* and *rna14*). Mutated sites are illustrated in figure 1*c*. In two independent mutant strains, a point mutation was found in the *ppr3* gene. In each mutant strain, temperature sensitivity was genetically confirmed to originate with a single mutation in the identified gene. Physiological functions and subcellular localization of each gene product were deduced from its description in the database, PomBase [17], and the results of comprehensive localization analysis [18]. Cells of all 8 mutant strains failed to form colonies on rich YES medium plates containing 3% glucose at the restrictive temperature (36°C), whereas they proliferated at the permissive temperature (26°C) (figure 1*a*).

**Table 1.** Mitochondrial *ts* strains. Identification of *ts* mutant genes is described in Material and methods. *ts*, temperature sensitive; *lgs*, low glucose sensitive; *shk*, super house-keeping.

phenotype	protein name	strain no.	mutation	presumed function	cell shape
*ts lgs shk*	Mrp4	77	E197K	mitochondrial ribosome subunit Mrp4/S2	normal
*ts lgs shk*	Mrp17	810	G31D	mitochondrial ribosome subunit Mrp17/S6	normal
*ts lgs shk*	Dmr1/Ppr3	506	W436*	mitochondrial PPR-repeat protein	normal
*ts lgs shk*	Dmr1/Ppr3	664	W174*	mitochondrial PPR-repeat protein	normal
*ts lgs shk*	Trz2	603	A623V	mitochondrial 3'-tRNA processing endonuclease	normal
*ts lgs shk*	Vrs2	642	W523*	mitochondrial valine-tRNA ligase	normal
*ts shk*	Cem1	424	G18E	3-oxoacyl-[acyl-carrier-protein]-synthase (condensing enzyme)	small, multiple septa
*ts shk*	Rna14	393	L271P	mRNA cleavage and polyadenylation specificity factor complex subunit	small, cut

### 2.2. The *lgs* phenotype of *ts* mitochondrial mutants

**Figure 2.**
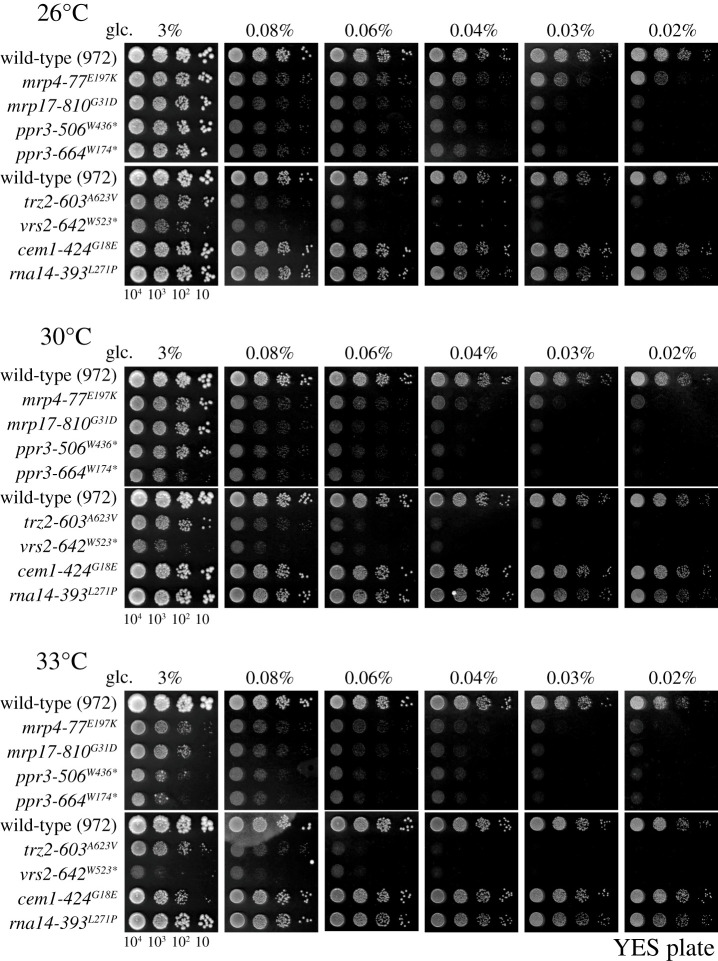
Six of eight mutants failed to proliferate under low-glucose conditions at the permissive (26°C) and semi-permissive temperatures (30 and 33°C). Spot tests were conducted to examine whether mutant cells could grow under low-glucose (0.02–0.08%) conditions. Cells of the wild-type (972) and cells of the indicated *ts* strains were serially diluted 10-fold and spotted on YES solid culture medium containing different concentrations of glucose (3–0.02%) and incubated at 26, 30 or 33°C for 3–4 days. See text for explanation of results.

Strains 77 and 810 were mutated in genes for mitochondrial ribosomal subunits, *mrp4*/S2 and *mrp17*/S6, respectively. The mutation sites were E197 K and G31D*,* respectively. These two strains are sensitive not only to high temperature (36°C) but also to low glucose. *mrp4-77* (E197 K) and *mrp17-810* (G31D) were unable to promote cell division at the permissive temperature in YES medium containing low glucose (0.08–0.02%), whereas they proliferated to form colonies on medium containing 3% glucose (figure 2). This low-glucose-sensitive phenotype (*lgs*) was also found in two *ppr3* mutants (506 and 664), the *trz2* mutant (603) and the *vrs2* mutant (642), even at 26°C, while proliferation of *ppr3*-*664* (W174stop) was also retarded on high-glucose medium (3% glucose) at 30 and 33°C, presumably due to severe temperature sensitivity (figure 2 and table 1). These two *ppr3* mutants are nonsense alleles (Trp codons encoding W436 and W174 changed to non-sense codons). Deletion of the *ppr3* gene was not lethal but caused a *ts* phenotype [13,18]. Ppr3 protein, which is located in mitochondria, contains 35-amino acid repeats (PPR, figure 1*c*) and belongs to a large family of RNA-binding proteins that are involved in post-transcriptional control of organelle gene expression [19,20]. While *ppr3* mutants exhibit increased levels of ROS (reactive oxygen species) [20], the reason why *ppr3* mutants produce the *lgs* phenotype is not understood. The *trz2-603* (A623V) mutant, which has been previously reported [15,21], is defective in mitochondrial tRNA processing endonuclease.

The *vrs2^+^* gene encodes a valine-tRNA ligase, and the *vrs2-642* (W523stop) mutant allele contains the nonsense mutation W523stop (the full length of the wild-type Vrs2 protein is 950 aa). The catalytic domain of valine-tRNA ligase is largely intact, while the C-terminal anti-codon binding domain is lost in this non-sense mutant. At the permissive temperature, the partially truncated mutant Vrs2 ligase might be sufficient for cells to grow. Alternatively, the other valine-tRNA ligase, Vrs1, might compensate for the lost function of the truncated Vrs2. *S. pombe* has two valine-tRNA ligase genes, *vrs1* and *vrs2*, which encode enzymes in the cytoplasm and in mitochondria, respectively [22]. Cytoplasmic Vrs1 may partly suppress the C-terminal region of *vrs2-642* (W523stop). In contrast to the mutants described above, *cem1-424* (G18E) and *rna14-393* (L271P) were not sensitive to low glucose. Thus, six of eight mitochondrial mutant strains showed the *lgs* phenotype.

Mitochondrial respiration may be necessary for *S. pombe* cells to divide efficiently in medium containing low glucose. Except Cem1, which is involved in lipid synthesis, other *ts* genes encode RNA-interacting proteins possibly involved in protein synthesis. Mrp4 and Mrp17 are ribosomal subunits, and Trz2 and Vrs2 are enzymes required for amino acyl tRNA synthesis. Deletion of the *ppr3* gene severely reduces levels of mitochondrial 15S-rRNA and proteins encoded by genes of the mitochondrial genome [19]. A deficiency of protein synthesis in mitochondria reportedly diminishes respiratory function; therefore, these mutations may cause defects in respiration, which is essential for cell proliferation in low glucose. Notably, although *rna14*-*393* (L271P) mutant cells were not sensitive to low glucose, Rna14 may also participate in gene expression (see discussion in the Opening Up section). In PomBase, this protein is inferred to localize in both the nucleus and mitochondria, based on the result of subcellular fractionation in *S. cerevisiae* [23], but direct evidence showing that *S. pombe* Rna14 functions in mitochondria has not been obtained.

### 2.3. Cellular morphology of eight *ts* mutants

Mutant strains were cultured in rich YES liquid culture medium containing 3% glucose at the restrictive temperature, 36°C, where they all failed to divide (figure 1*b*). Cellular morphology of *mrp4-77* (E197 K) and *mrp17-810* (G31D) mutants at the restrictive temperature appeared normal in rich culture medium. They are rod shaped, similar to wild-type cells (figure 1*b*). Budding yeast remains viable, despite deletion of the homologous MRP4, while the gene-disrupted strain of fission yeast, *Δmrp4*, is inviable (SGD (www.yeastgenome.org) and PomBase). Similarly, the *ppr3*-*506* (W436stop) mutant yielded rod-shaped cells at 36°C. Two other strains, *trz2-603* (A623 V) and *vrs2-642* (W523stop), also showed normal looking, rod-shaped cells. By contrast, *cem1-424* (G18E) and *rna14-393* (L271P) produced aberrantly shaped cells at 36°C. Most *cem1* mutant cells were septated and contained two daughter nuclei, suggesting that cytokinesis may be blocked in this mutant. In *rna14* mutant cells, nuclei were often displaced in only one of the daughter cells during cytokinesis and/or the cell was ‘cut' by a septum. These results are consistent with findings of Sonkar *et al.* [24] that the defective *rna14-11* (R316Q) mutant caused impaired cell cycle progression and genomic instability, leading to chromosome mis-segregation.

### 2.4. The membrane potential and morphology of mitochondria in *ts* mutants

We then examined whether these eight mutations affect the mitochondrial inner membrane potential. To estimate the mitochondrial potential, cells were stained with a fluorescent dye, MitoTracker Red CMXRos, which accumulates in mitochondria in a manner dependent on the membrane potential according to the manufacturer. After staining with MitoTracker Red CMXRos, fluorescence intensity of cells was measured by flow cytometry.

**Figure 3.**
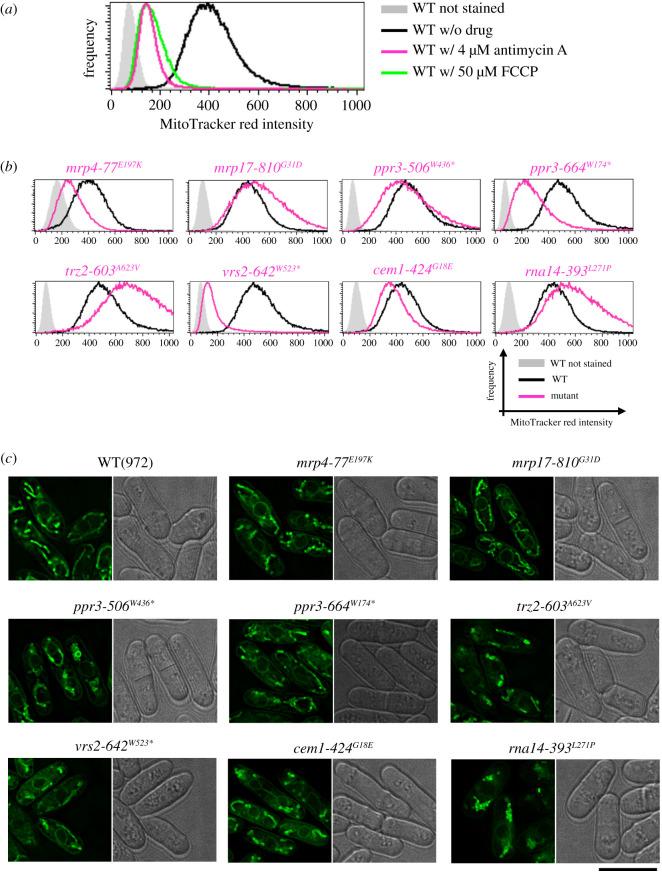
Mitochondrial membrane potential and morphology of *ts* mutants. (*a*) Wild-type cells treated with 4 µM Antimycin A or 50 µM FCCP were stained with MitoTracker Red CMXRos, a fluorescent dye sensitive to the mitochondrial membrane potential, and fluorescence intensity was measured by flow cytometry. Measurements in antimycin A-treated cells and those with FCCP are shown in magenta and green, respectively. Cells without any drug treatment were used as positive controls (black). As a negative control to estimate background fluorescence due to cellular autofluorescence, cells not stained with MitoTracker Red were used (grey). (*b*) Indicated mutant cells cultured in EMM2 liquid medium at 36°C were stained with MitoTracker Red CMXRos, and fluorescence intensity was measured by flow cytometry. In each batch, wild-type cells with and without MitoTracker Red staining were employed as positive and negative controls, respectively. Measurements in the mutants, the positive control, and the negative control are shown in magenta, black, and gray, respectively. (*c*) Wild-type and indicated mutant cells were cultivated in EMM2 medium at 36°C and stained with a membrane-potential-insensitive fluorescent dye, MitoTracker Green FM. Fluorescence and bright field images of cells were taken using a DeltaVision Elite high-resolution microscope. Scale bar, 10 µm.

To confirm that intensity of MitoTracker Red fluorescence indeed reflects the mitochondrial membrane potential, we first tested wild-type (972) cells treated with 4 µM antimycin A or 50 µM carbonyl cyanide 4-(trifluoromethoxy)phenylhydrazone (FCCP). Antimycin A inhibits cytochrome b reductase in the electron transfer chain (ETC) producing the membrane potential, whereas FCCP eliminates the membrane potential by transporting protons across the mitochondrial inner membrane (figure 3*a*). Treatment with these mitochondrial poisons reduced fluorescence intensity to nearly background levels, indicating that the mitochondrial membrane potential can be estimated from MitoTracker Red fluorescence intensity.

Mutant cells cultured in EMM2 minimal medium at 36°C for 4 h were stained with MitoTracker Red CMXRos, and flow cytometry results are shown in figure 3*b*. Contrary to expectation, not all mutations diminished the potential, although all mutant cells ceased to proliferate in this condition. *mpr4-77* (E197 K), *ppr3-664* (W174stop) and *vrs2-642* (W523stop) mutations greatly reduced the mitochondrial potential. *ppr3-506* (W436stop) and *cem1-424* (G18E) mutations also appeared to reduce the potential slightly. Other mutations, however, did not decrease the mitochondrial potential. Especially in *trz2-603* (A623 V) mutant cells, MitoTracker Red fluorescence was clearly stronger than in wild-type cells. This result appears consistent with a previous report showing that over-production of Trz2 protein reduces mitochondrial potential [25] and implies that dysfunction of Trz2 may not affect ETC activity, but may impair ATP generation, which consumes energy of the mitochondrial membrane potential.

Mitochondrial morphology in these *ts* mutants was examined (figure 3*c*). Mutant cells cultivated at 36°C for 4 h were stained with MitoTracker Green FM. Unlike MitoTracker Red CMRos used above, this fluorescent dye accumulates and becomes fluorescent in mitochondria in a manner not dependent on the potential, while it tends to stain other cellular structures non-specifically, according to the manufacturer. In all mutants except *rna14-393* (L271P), fibrous mitochondria similar to those in wild-type cells were observed, while the quantity (volume and/or number) of mitochondria appeared reduced in *mpr4-77* (E197K), *trz2-603* (A623V) and *vrs2-642* (W523stop) mutants. In *rna14-393* (L271P), mitochondria aggregated and formed blob-like structure in the cytoplasm. Similar blob-like mitochondria were observed also in *ppr3-506* (W436stop) mutant cells.

### 2.5. All mutants showed a super-housekeeping (*shk)*-defective phenotype

**Figure 4.**
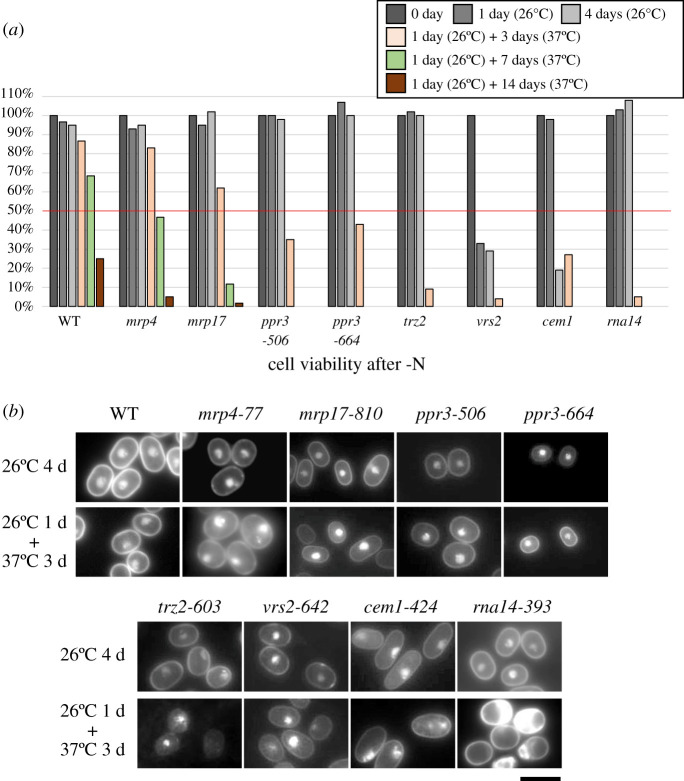
All mutants show super-housekeeping (*shk)*-defective phenotypes. (*a*) Viability of mutant cells (%) was determined after cultivation in nitrogen-deficient EMM2 (EMM2-N) medium for 0, 1, and 4 days at 26°C or for 1 day at 26°C, followed by 3 extra days at 37°C. For *mrp4*, *mrp17* and wild-type, cell viability after prolonged incubation (7 and 14 days) at 37°C was also measured. For measurement of viability, 300 cell bodies were plated on YPD rich medium and incubated at the permissive temperature (26°C). Cell viability was calculated from numbers of colonies formed, and shown as a percentage. (*b*) Cellular and nuclear morphologies of the indicated *ts* mutant strains were examined after cultivation for 4 days at 26°C (upper panels) and for 1 day at 26°C and extra 3 days at 37°C (lower panels) in EMM2-N liquid medium at the restrictive temperature. Cells were fixed with 2.5% glutaraldehyde and stained with DAPI before fluorescence microscopy. Scale bar, 10 µm.

Another nutritional response examined was a loss of viability when the nitrogen source (NH_4_Cl) was eliminated from the synthetic EMM2 culture medium. Under this condition in quiescence, wild-type cells recycle intracellular nitrogen sources to maintain high viability for a long period (approx. one month) [26]. Cell viability (mitotic competence, MC [15]) of four *ts* strains, *trz2*, *vrs2*, *cem1* and *rna14* mutant strains was greatly reduced after 3 days at 37°C in nitrogen-deficient EMM2 (EMM2-N) cellular quiescence medium (figure 4*a*). Two *ppr3* mutants also lost viability significantly after 3 days at 37°C in EMM2-N medium. While 90–100% of wild-type cells maintained the ability to form colonies upon re-addition of nitrogen during incubation in quiescence medium, mutant strains failed to recover colony formation ability upon nitrogen replenishment. As these genes are required for cell survival in both vegetative and quiescent conditions, they were collectively designated as *shk* (super-housekeeping) genes [15]. Two mutant strains defective in ribosomal subunits, *mrp4* and *mrp17*, maintained relatively high viability up to 3 days in comparison to six other mutants, but eventually lost viability significantly after more than one week of cultivation in EMM2-N medium, indicating that all seven of these genes are required for viability in both proliferative and quiescent conditions.

*ppr3*, *trz2* and *vrs2* mutants, as well as the *mrp4* mutant, exhibited a round cell shape, which is normal for quiescent cells (figure 4*b*). In contrast to these mutants, cells of *rna14-393* (L271P) showed abnormal morphology in nitrogen-starved, G0-quiescent conditions at the restrictive temperature, as well as in vegetative conditions (figure 1*b*). Cells were often bisected with one half lacking a nucleus. Aberrant septation was observed in small cells in nitrogen-deficient culture medium, suggesting that cells became physically inviable during colony formation or during entry into G0 phase (figure 4*b*).

### 2.6. *cem1-424* (G18E), defective in fatty acid synthesis, is also an *shk* mutant

*cem1-424* (G18E) is mutated in the condensing enzyme (3-oxoacyl-[acyl-carrier-protein]-synthase), which is involved in fatty acid synthesis [27]. The mutation seemed unrelated to RNA metabolism. Fission yeast Cem1 protein is located in mitochondria [18]. Budding yeast CEM1 is implicated in respiration as well as in lipid metabolism, but is non-essential for cell viability (SGD [28]). The mutation of fission yeast *cem1-424* (G18E) is located at the N-terminus of the thiolase domain. This mutant is not only *ts*, but also *shk*, losing viability in quiescence in media without nitrogen. In the nitrogen starved G0 phase, cells were ellipsoidal instead of round, and nuclei were abnormally condensed (figure 4*b*). It may be noteworthy that the *ts* phenotype of *cem1-424* (G18E) was reportedly suppressed in medium containing rapamycin [29], an inhibitor of the TOR enzyme complex, which is sensitive to nutritional levels. Cem1 may be one of many gene products that respond to TOR signalling.

### 2.7. Identified genes are a small fraction of the many essential genes encoding mitochondrial proteins

**Figure 5.**
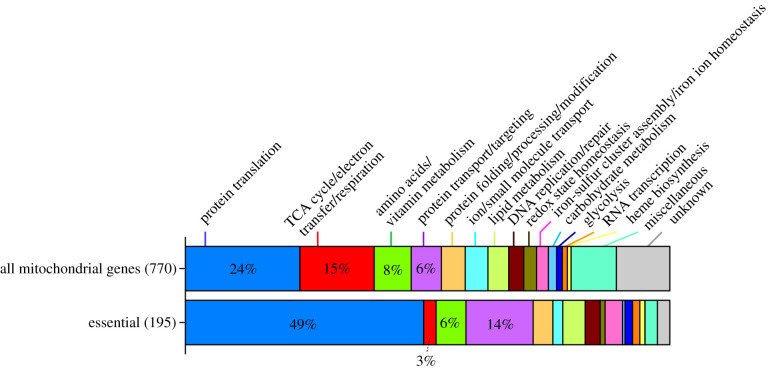
Classification of *S. pombe* mitochondrial protein genes. The *S. pombe* genome contains 770 genes encoding putative mitochondrial proteins [17,18]. Gene disruption studies showed that 195 are absolutely essential for cell viability. They are functionally classified into 16 sub-groups: protein translation, protein transport/targeting, protein folding/processing/modification, RNA transcription, DNA replication/ repair, iron-sulfur cluster assembly/ iron ion homeostasis, heme biosynthesis, amino acids/vitamin metabolism, lipid metabolism, glycolysis, TCA cycle/ electron transfer/respiration, redox state homeostasis, ion and small molecule transport, carbohydrate metabolism, and miscellaneous and unknown functions. The majority of essential mitochondrial genes are involved in translation (49%) and protein transport/targeting (14%).

According to a genome-wide gene disruption study [13] and PomBase (as of 26 January 2021), 1222 genes of *S. pombe* are required for vegetative cell proliferation in complete medium. Previous studies and annotation in PomBase indicated that 770 gene products are known or predicted to locate in mitochondria ([18] and PomBase). Combining these information, about 25% of 770 genes (195 genes; 195/770 = 0.253) are supposed to encode essential mitochondrial proteins, loss of which causes cell lethality. The presumed functions of proteins located in mitochondria include (1) protein translation, (2) TCA cycle/electron transfer/respiration, (3) amino acid/vitamin metabolism, (4) protein transport/targeting, (5) ion/small molecule transport, (6) iron-sulfur cluster assembly/iron ion homeostasis (required for enzymatic centers of many proteins) and (7) protein folding/processing/modification (figure 5). 24% and 15% of mitochondrial proteins are involved in protein translation and respiration, respectively, whereas only 6% of them function in protein transport/targeting. By contrast, the great majority of the 195 essential mitochondrial proteins have one of two functions, mitochondrial protein translation (49%) or mitochondrial protein transport/targeting (14%) (figure 5; electronic supplementary material, table S1). Only 3% of essential proteins are related to respiration. Thus, proteins related to translation and transport/trafficking are enriched among essential proteins, whereas those related to respiration are underrepresented, suggesting that mitochondria are essential as centers for protein regulation (i.e. protein expression and targeting). In regard to fission yeast cell viability, respiration is comparatively less important. Consistently, while the total number of *ts* mutations identified in this study was relatively small, most of the identified mutants are predicted to impair mitochondrial protein translation (transcription, tRNA maturation and mitochondrial ribosome biogenesis).

## 3. Discussion

In this study, gene identification and basic characterizations of eight mitochondrial *ts* mutants of fission yeast, defective in mitochondrial translation, RNA metabolism, and lipid synthesis, were conducted. While two strains (*ppr3-506* (W436stop) and *trz2-603* (A623V)) were partly described previously [15,21], six others (*mrp4-77* (E197K)*, mrp17-810* (G31D)*, ppr3-664* (W174stop)*, vrs2-642* (W523stop)*, cem1-424* (G18E)*, rna14-393* (L271P)) are newly reported here. Two alleles were obtained from the same *ppr3*^+^ gene. Among the seven genes identified, six (*mrp4^+^, mrp17^+^, trz2^+^, vrs2^+^, cem1^+^*, *rna14^+^*) were absolutely essential for cell viability (i.e. gene disruption led to cell lethality, but not temperature sensitivity, even under nutrient-rich vegetative conditions). Notably, the genes analysed in this study represent only a tiny fraction of the 195 essential mitochondrial proteins, the great majority of which have scarcely been studied. Conditional mutants are useful for understanding essential gene functions. Reverse-genetic engineering approaches, such as fusion with auxin degron [30,31], might be useful to study the remaining essential mitochondrial proteins.

Interestingly, all eight *ts* mutants isolated proved responsive to nutritional stress. All lost viability during G0 quiescence (the *shk* phenotype), under nitrogen deprivation. Six of eight mitochondrial mutants also showed the *lgs* phenotype. Two *lgs* mutants, *mrp4-77* (E197K) and *mrp17-810* (G31D), defective in ribosome subunits S2 and S6, respectively, cannot proliferate, even at the permissive temperature (26°C), in low glucose. These mutations are supposed to result in inefficient translation of 11 mitochondrial proteins encoded by the small mitochondrial genome, and consequently cause the *lgs* phenotype. Thus, these two *ts* strains will be useful to examine the effect of mitochondrial ribosome synthesis under different physiological conditions. The mutation in the *mrp17-810* (G31D) allele causes a single amino acid substitution from G to D in the conserved domain of the ribosomal S6 subunit, resulting in the *ts* and *lgs* phenotypes (figure 1*c*). The same substitution might be applied to higher eukaryotic orthologues by altering the chromosomal gene (MRPS6 HGNC:14051), and the phenotype of such a mitochondrial mutant, if obtained, will be of certain value for understanding the phenotype. Four other strains, *ppr3-506* (W174stop), *ppr3-664* (W436stop), *trz2-603* (A623 V) and *vrs2-642* (W523stop), involved in mitochondrial RNA metabolism and probably also in protein synthesis, produced the same *lgs* and *shk* phenotypes. Thus, defects in mitochondrial protein translation resulted in a distinct inability to use dilute carbon sources and to recycle intracellular nitrogen sources in G0 quiescent cells cultured without NH_4_Cl in the medium.

Two other mutants, *cem1-424* (G18E) and *rna14-393* (L271P), exhibited the *shk* phenotype, although they were not sensitive to low glucose. Thus, all isolated mutants may lose mitotic competence during G0 phase, or alternatively, may lose the capacity to exit from G0 phase upon replenishment of nitrogen. It is not surprising that mitochondria become essential under nitrogen starvation, as amino acid metabolism is quite active in mitochondria and mitochondrial respiration is important for synthesis of certain amino acids, such as arginine [11]. However, since none of the defective genes examined in this study are directly related to nutrient recycling, and since during nitrogen-depleted G0 phase, amino acid levels are thought to be maintained by recycling rather than synthesis, further studies are required to understand the mechanisms underlying the nutritional phenotypes. Future studies may also reveal how many essential mitochondrial proteins are required for nitrogen recycling to enable cells to sustain cellular quiescence under nitrogen starvation for long periods. Interestingly, the *cem1-424* (G18E) mutant is reportedly rescued by addition of rapamycin [29]. Hence this mutant showed multiple phenotypes: *shk* phenotype, abnormal lipid synthesis, abnormal septation under nitrogen starvation and the rescue of the *ts* phenotype by rapamycin. Rapamycin inhibits the protein kinase activity of TORC1 (Target Of Rapamycin Complex 1), and TORC1 is responsive to nutritional cues [32]. TORC1 activity is downregulated upon nitrogen starvation. Thus, inhibition of TORC1 by rapamycin or nitrogen starvation may enhance Cem1 enzymatic activity, which may promote intracellular recycling of nitrogen in nitrogen-depleted environments.

Since all *ts* mutants investigated in this study presented nutritional, as well as *ts* phenotypes, such nutritional phenotypes may be common for mutants of essential genes encoding mitochondrial proteins. Construction of a full set of conditional-lethal mutants in this category of genes encoding mitochondrial proteins, although time-consuming, would reveal novel aspects of diverse mitochondrial functions, judging from the broad range of the 195 essential genes for mitochondrial proteins (electronic supplementary material, table S1). Note that most of these mitochondrial proteins are conserved in mammals. Considering that mitochondria are essential organelles in the brain and muscle, and also for human longevity, the study of mitochondria using a fission yeast model promises potentially large dividends in understanding mitochondrial roles in humans.

## 4. Opening up

As discussed above, all identified genes except *rna14* and *cem1* are involved in mitochondrial gene translation, implying that expression of proteins encoded in the mitochondrial genome is essential for cell viability. While mitochondrial localization of Rna14 has not been shown directly in *S. pombe*, abnormal mitochondrial morphology/distribution in *rna14-393* (L271P) suggests its mitochondrial role (figure 3*c*). Rna14 is reportedly involved in mRNA cleavage and polyadenylation reactions, although mRNAs transcribed from the mitochondrial genome are unlikely to be polyadenylated. This protein may be involved in processing/polyadenylation of non-coding RNAs transcribed from the mitochondrial genome, such as mitochondrial small RNAs, which were recently identified by mitochondrial transcriptome analysis [33]. Cem1 is involved in mitochondrial fatty acid synthesis. Unlike fatty acid synthesis in the cytoplasm, in which multiple steps of fatty acid synthesis reaction are catalysed by a single multifunctional enzyme complex composed of *α* and *β* subunits, fatty acid synthetase [34,35], each step of mitochondrial fatty acid synthesis is catalysed by separate enzymes, including Cem1 [36]. Unlike Cem1, other enzymes required for mitochondrial fatty acid synthesis, such as Mct1, Htd2, Etr1 and Oar1, are not essential for cell viability in *S. pombe*, according to PomBase. Reasons for this apparent discrepancy remain unknown. Cem1 may have a cryptic function that is not related to fatty acid synthesis, but is required for mitochondrial gene expression.

Unlike petite positive budding yeast, mitochondrial DNA is indispensable for survival in petite negative *S. pombe*. While all protein-coding genes in the mitochondrial genome, except *rps3*, which encodes a subunit of mitochondrial ribosomes, are involved in respiration, inhibition of mitochondrial respiration itself by drugs or mutations did not result in cell death. So, it is enigmatic that mutations diminishing gene expression from the mitochondrial genome cause lethality in petite negative yeasts. In another petite negative yeast, *Kluyveromyces lactis*, complete loss of the mitochondrial potential (which is required for not only respiration, but also other vital reactions) is proposed to be a cause of cell death upon loss of the mitochondrial genome [37]. In this organism, inhibition of respiration alone does not completely eliminate the membrane potential, which appears to be maintained by reverse reaction of ATP synthesis by F0-F1 ATP synthetase under respiration-defective conditions [37]. However, in *S. pombe*, inhibition of respiration with Antimycin A alone eliminated the mitochondrial membrane potential as nearly completely as with FCCP treatment (figure 3*a*). Additionally, not all eight *ts* mutations caused reduction of the mitochondrial membrane potential at a restrictive temperature, although all mutants prevented cell division. Thus, cell death in these mutants is probably not caused by loss of the membrane potential. Defects in amino acid synthesis are also unlikely to be the cause, as *ts* mutants failed to proliferate even in YES rich medium at a restrictive temperature (figure 1*a*). Proteomic and metabolomic analyses of identified mutants may eventually explain why *S. pombe* is petite negative.

## 5. Material and methods

### 5.1. General techniques and strains

General procedures for handling *S. pombe* have been described previously [38]. For cultivation of *S. pombe* cells, rich yeast extract/glucose/supplement (YES) medium and synthetic minimal EMM2 medium were used with modified glucose concentrations, as indicated [39]. Isolated *ts* mutant strains were backcrossed at least once, and the *ts* phenotype was confirmed to be caused by a single gene mutation in each strain. Unless otherwise stated, cells were cultivated at 26°C. Cell viability was expressed as the ratio of the number of colonies formed on YPD solid medium containing 111 mM (2%) glucose to the total number (300) of cell bodies plated.

### 5.2. Fluorescence microscopy

Fluorescence microscopy was performed using Axiovert 200M and Axioplan 2 microscope systems (Carl Zeiss, Oberkochen, Germany) equipped with 100 × objective lenses (NA 1.40). 4',6-diamidino-2-phenylindole (DAPI, 50 µg/ml) was applied to cells fixed with 2.5% glutaraldehyde [40] for fluorescent staining of DNA. For staining of mitochondria, 200 nM MitoTracker Green FM was added to culture medium and cells were cultivated for 0.5 h before observation. High-resolution images of mitochondria were obtained using a DeltaVision Elite system (Cytiva, Marlborough, MA, USA).

### 5.3. Mitochondrial membrane-potential measurement

Wild-type and mutant cells were cultivated in EMM2 medium at 36°C for 3.5 h. Cells were cultivated further for 0.5 h after addition of 50 nM MitoTracker Red CMXRos to the medium. Cell culture was then diluted with two volumes of phosphate-buffered saline and kept on ice. Fluorescence intensity of each cell was measured with Flow cytometry. Wild-type cells to which MitoTracker Red was not added were used as a negative control. To examine effects of antimycin A (final concentration: 4 µM) or FCCP (final concentration: 50 µM), each drug was added to the medium 0.5 h before addition of MitoTracker Red.

## Data accessibility

This article has no additional data.
